# A Case of Cocaine-Induced Uvular Edema

**DOI:** 10.7759/cureus.12308

**Published:** 2020-12-26

**Authors:** Matthew Heard, Kathleen McMahon, Thomas Nappe

**Affiliations:** 1 Emergency Medicine, St. Luke's University Health Network, Bethlehem, USA; 2 Emergency Medicine, St. Luke’s University Health Network, Bethlehem, USA; 3 Medical Toxicology, St. Luke's University Health Network, Bethlehem, USA

**Keywords:** uvular edema, quinke’s disease, cocaine

## Abstract

A 26-year-old male presented to the emergency department with a chief complaint of globus sensation and sore throat in the setting of recent cocaine use. On physical examination, he was found to have isolated uvular edema and erythema. After excluding other potential inciting mechanisms of Quincke’s disease, he was treated with anti-inflammatory medication and was observed for any development of airway compromise prior to discharge.

## Introduction

Isolated uvular edema is often referred to as Quincke’s disease. Owing to a variety of etiologies, it can present as an emergent condition threatening airway patency. Rapid assessment for potential airway compromise is the primary focus of initial management in addition to the identification of characteristic physical examination findings. An accurate account of the inciting mechanism, through good history taking, is essential in determining effective treatment.

## Case presentation

**Figure 1:**
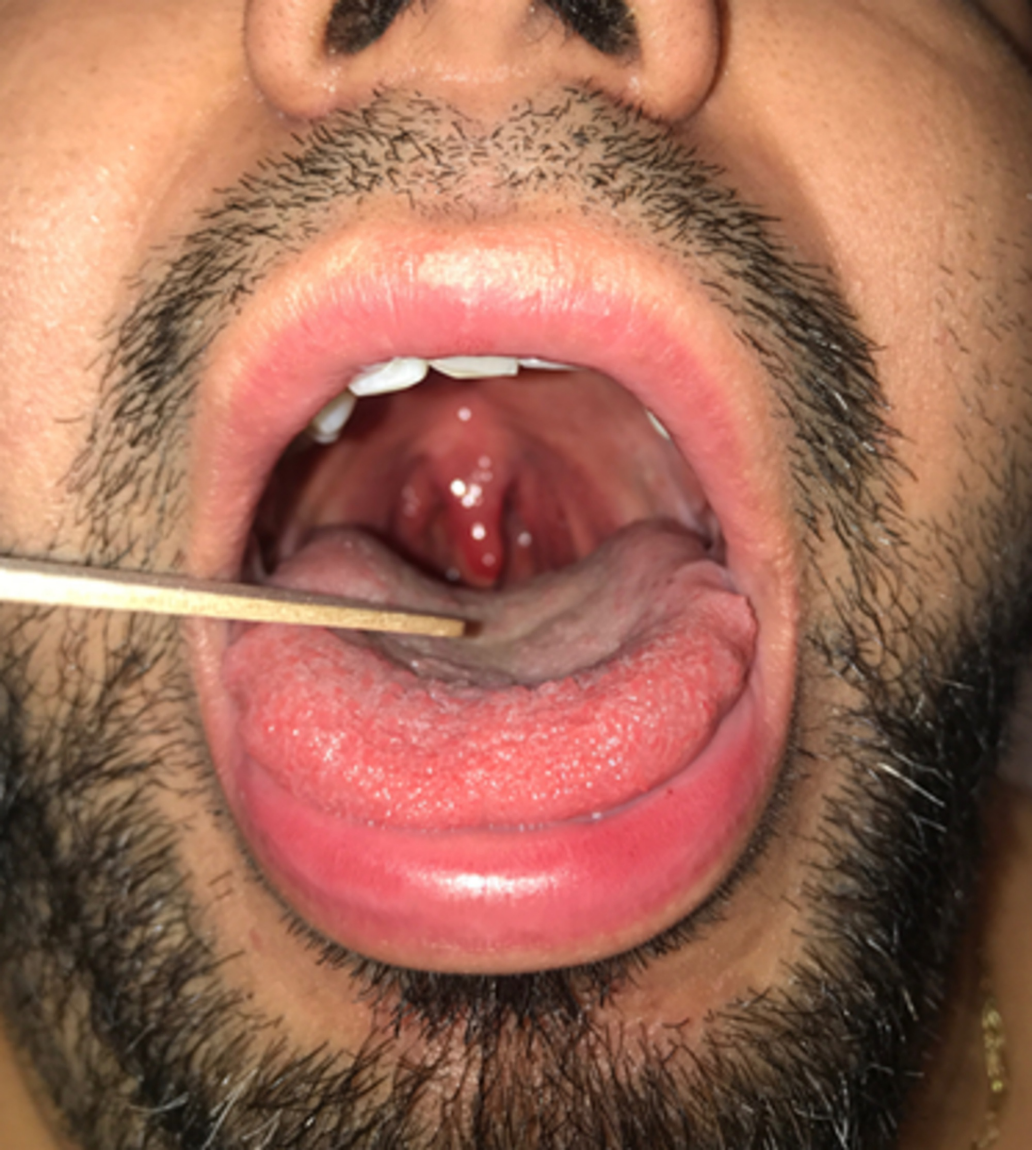
Isolated uvular edema and erythema consistent with Quincke’s disease.

A 26-year-old male presented to the ED with a one-day history of globus sensation and “throat inflammation.” He admitted to binge drinking the previous evening and snorting cocaine 12 hours prior to presentation. He denied dysphagia, odynophagia, dysphonia, dyspnea, dyspepsia, rash, known allergies, or prior similar episodes. He denied fever, chills, cough, or recent illness. On physical examination, he was afebrile, mildly tachycardic, and breathing comfortably without stridor or wheeze. The posterior oropharynx was noted to be erythematous without exudate, while the uvula was swollen and erythematous (Figure 1). No cervical lymphadenopathy was appreciated. His tachycardia resolved with one liter of normal saline, and his symptoms improved with intravenous ketorolac and dexamethasone. He was monitored for signs of airway compromise for several hours. After a successful PO challenge, he was discharged with a four-day steroid burst and strict return precautions.

## Discussion

Quincke’s disease was first described in the late 1800s as angioneurotic edema of the uvula. It is generally thought to be the result of increased vascular permeability [1]. While in many cases idiopathic, it may result from a broad range of etiologies including allergic IgE-mediated mast cell degranulation, anaphylactoid reaction (e.g., from radiocontrast media), infection, hereditary angioedema, local thermal or toxic reactions (e.g., from cocaine, marijuana, or general anesthesia), neoplastic processes, and even snoring. Iatrogenic causes include trauma secondary to endotracheal intubation or endoscopy, as well as reactions to medications, in particular ACE-inhibitors, anesthetics, and substances such as aspirin that interfere with arachidonic acid metabolism [2]. The likely causative mechanism is important as it helps dictate the most appropriate treatment. Of course, signs of airway collapse or respiratory distress may limit history-taking. While uvular edema itself can obstruct the airway, the patient should be evaluated for concomitant hypopharyngeal swelling. Immediate treatment including securing the patient’s airway may be required.

## Conclusions

Isolated uvular edema, or Quincke's disease, has several etiologies including cocaine use. Depending on the inciting agent and severity of edema, treatment may range from supportive care with anti-inflammatory and steroid medication to endotracheal intubation in the most severe cases. Identification of this finding on physical examination in the context of historical elements of the presentation can significantly narrow the differential diagnosis for a patient with an oropharyngeal complaint. Awareness of possible inciting agents is essential. Prognosis is generally good, and many patients can be discharged after treatment and observation in the emergency department.
